# Psychometric Evaluation of the Parent Effort Scale

**DOI:** 10.3389/fresc.2022.780302

**Published:** 2022-02-02

**Authors:** Katherine B. Bevans, Taye M. Hallock, Aimee Piller, Beth Pfeiffer

**Affiliations:** ^1^Janssen Pharmaceuticals Inc., Horsham, PA, United States; ^2^Department of Health and Rehabilitation Sciences, Temple University College of Public Health, Philadelphia, PA, United States; ^3^Piller Child Development, LLC, Phoenix, AZ, United States

**Keywords:** measurement, autism spectrum disorder (ASD), parent effort, pediatric, psychometric

## Abstract

**Objective:**

The Parent Effort Scale (PES) is a parent report questionnaire designed to quantify the level of effort required of caregivers to assist their children in developmentally appropriate home- and community-based activities. This manuscript describes the psychometric evaluation of the PES.

**Method:**

Data collected from 304 parents of children ages 2–7 years (167 parents of a children with autism spectrum disorder and 137 parents of neurotypical children) were factor analyzed, calibrated using item response theory, and evaluated for construct validity.

**Results:**

The final PES scales are reliable and valid measures of the level of parental effort required to assist children in dressing, personal hygiene, sleep, socialization at home, participation in community events, and access to healthcare. A total score reflects overall parental effort.

**Conclusion:**

The PES can be used to plan and evaluate the effectiveness of interventions that aim to help parents enhance children's participation opportunities and thus, support their cognitive and social development.

## Introduction

Participation in home and community contexts is essential for children's cognitive, social, and behavioral skill development, relationships, and health-related quality of life (1–4). Preschool-aged children with autism spectrum disorder (ASD) participate in fewer self-care, community mobility, leisure, social interaction, domestic chore, and education activities than their neurotypical peers (5). For these children, participation opportunities are restricted by sensory sensitivities and social, communication, and behavioral difficulties, which are often elicited or exacerbated by task demands and triggers due to a mismatch between the child and their environment (5–7).

Parents of young children, especially those with ASD and other neurodevelopmental conditions, use a variety of strategies to enable their child's successful participation in home- and community-based activities (8, 9). Strategies include strict adherence to set routines, constant supervision, use of repeated verbal and physical prompts to support task completion, reward or punishment systems, modifying the environment to limit exposure to aversive stimuli, removing children from challenging situations, and comforting children when they become distressed (9). These strategies are designed to improve child-environment fit. However, children's behavior challenges may continue despite parents' use of multiple caregiving strategies. Persistent behavioral challenges may erode parents' confidence and thwart future attempts to facilitate participation, further limiting children's opportunities for social learning as well as child and family quality of life (10).

Parents of children with ASD describe having to “pick their battles” when considering how to balance children's participation with their capacity to manage problem behaviors (10). They may abandon or delay task demands or participation attempts if problem behaviors outweigh their energy and ability to manage them (10). Pfeiffer et al. observed a decision-making process wherein parents of children with ASD considered the amount of effort needed to support their child's participation in relation to the meaningfulness of the activity (11). Parents restricted children's participation in non-essential or non-meaningful activities when they deemed it to be too “effortful” (11). Although parent effort significantly influences the quality of family life and opportunities for children to engage in activities that are essential for their development, the concept is under-represented in existing measurement tools. A measure of parent effort could be used to identify caregivers who are either expending very high (unsustainable) levels of effort or avoiding activities with excessive effort demands. Providers could use the measure to target skill-building interventions or environmental modifications that improve environmental fit and reduce parent effort demands, ultimately enhancing children's participation opportunities.

The Parent Effort Scale (PES) measures the amount of parent effort required to enable children's participation in common developmentally appropriate home- and community-based activities. Instrument content was informed by 34 parent/caregiver interviews regarding caregiver strategies for enhancing children's participation at home and in the community (11). All participants were full time caregivers of children ages 3–7 years with ASD. Initial versions of the PES items were generated based on interview results and thereafter, items were refined based on cognitive interviews conducted with parents of children with ASD (12). Here, we describe further refinement and validation of the PES through psychometric evaluation.

## Methods

All study procedures were approved by the Institutional Review Board at Temple University.

### Participants

Participants were 304 parents of children ages 2 years to 7 years (*M* = 4.2, *SD* = 0.9; Table 1). Participants were purposively sampled to represent parents of children with ASD (*n* = 167) and neuro-typical development (*n* = 137). Parents were recruited by collaborating schools and via ASD support groups and social media sites.

**Table 1:** Child and family characteristics.

	**Total sample**	**ASD group**	**Neuro-typical group**
*N*	304	167	137
Child age, years: M (SD)	4.2 (0.9)	4.2 (1.0)	4.3 (0.9)
Child gender: *n*, % male	199, 65%	127, 76%	72, 53%
Child race/ethnicity: *n*, %			
White/Caucasian	243, 80%	123, 74%	120, 88%
Black/African-American	12, 4%	11, 7%	1, 1%
Hispanic/Latino	12, 4%	11, 7%	1, 1%
Other	37, 12%	22, 13%	15, 11%
Family annual income: *n*, %			
< $19,999	16, 5%	15, 9%	1, 1%
$20,000–$39,999	37, 12%	33, 20%	4, 3%
$40,000–$59,999	26, 9%	17, 10%	9, 7%
$60,000–$79,999	44, 14%	24, 14%	20, 15%
$80,000–$99,999	57, 19%	29, 17%	28, 20%
≥$100,000	121, 40%	46, 28%	75, 55%
Language Spoken at Home: *n*, % English	293, 96%	163, 98%	130, 95%
Residential community type: *n*, %			
Major urban	78, 26%	43, 26%	35, 26%
Suburban	160, 53%	79, 47%	81, 59%
Small town	46, 15%	31, 19%	15, 11%
Rural	20, 7%	14, 8%	6, 4%

### Measures

#### Child/Family Characteristics

Parents provided sociodemographic information including child age, gender, race/ethnicity, family income, residential community type (urban, suburban, small town, rural), and the primary language spoken at home.

#### ASD Symptoms

Parents completed the Gilliam Autism Rating Scale–3rd edition (GARS-3), a reliable and valid norm-referenced informant-report screener for ASD (13). A total Autism Index score characterizes children's restricted/repetitive behavior, social interaction, social community, and emotional response for all children, plus cognitive style and maladaptive speech for non-verbal children. The GARS-3 Autism Index score > 70 was used to classify children into ASD and neuro-typical subgroups.

#### Parent Effort Scale

Parents completed 34 PES items, each describing a home- or community-based activity (Table 2). For each, parents are asked “how much effort is it for YOU to help your child participate in this activity?” All items use a 5-point Likert scale with lower score indicating less parental effort: (1) none, (2) a little, (3) some, (4) a lot, (5) too much to participate.

**Table 2:** Parent Effort Scale (PES) item response frequencies, *n* (%).

	**ASD**					**Neurotypical**				***X^2^***
	**1**	**2**	**3**	**4**	**5**	**2**	**3**	**4**	**5**	
**Dressing scale**										
Dressing (excluding socks/shoes/coat)	8 (5)	39 (26)	55 (36)	43 (28)	6 (4)	57 (43)	21 (16)	4 (3)	0 (0)	86.48^*^
Putting on socks/shoes	14 (9)	36 (24)	45 (30)	51 (34)	4 (3)	60 (45)	18 (14)	6 (5)	00 (0)	74.90^*^
Putting on coat by self or another	19 (13)	35 (23)	52 (34)	39 (26)	6 (4)	43 (33)	13 (10)	2 (2)	0 (0)	95.29^*^
**Hygiene/self-care scale**										
Tooth brushing	12 (8)	35 (24)	30 (20)	59 (40)	12 (8)	62 (47)	26 (20)	10 (8)	1 (1)	60.18^*^
Bathing (excluding washing hair)	16 (11)	34 (21)	34 (23)	57 (38)	13 (9)	48 (36)	36 (27)	15 (11)	0 (0)	46.05^*^
Washing hair	11 (7)	18 (12)	34 (23)	63 (43)	22 (15)	46 (35)	35 (27)	35 (27)	5 (4)	30.09^*^
Toileting (diaper changes/using toilet)	19 (13)	40 (26)	35 (23)	45 (30)	12 (8)	53 (40)	17 (13)	7 (5)	0 (0)	64.35^*^
**Sleep scale**										
Falling asleep	37 (25)	28 (19)	47 (31)	32 (21)	6 (4)	45 (34)	12 (9)	12 (9)	2 (2)	40.71^*^
Staying asleep through night	46 (30)	39 (26)	31 (21)	24 (16)	11 (7)	41 (31)	13 (10)	5 (4)	1 (1)	32.80^*^
**Home social scale**										
Eat	35 (23)	41 (28)	38 (26)	32 (21)	3 (2)	41 (39)	13 (5)	5 (4)	1 (1)	54.79^*^
Mealtime with family members	26 (17)	39 (26)	41 (27)	31 (21)	13 (9)	44 (33)	9 (7)	6 (5)	0 (0)	72.13^*^
Play with other children in home	29 (19)	28 (19)	46 (30)	39 (26)	9 (6)	31 (23)	2 (2)	3 (2)	1 (1)	112.10^*^
Play with toys/objects	53 (35)	27 (18)	38 (25)	27 (18)	5 (3)	19 (14)	2 (2)	2 (2)	0 (0)	78.87^*^
**Community participation**										
parties for another child in community/at another's home	8 (5)	20 (13)	46 (30)	52 (34)	25 (17)	61 (46)	13 (10)	1 (1)	0 (0)	149.62^*^
meals at family or friends' home	11 (7)	26 (17)	49 (32)	48 (32)	17 (11)	57 (43)	17 (13)	2 (2)	0 (0)	115.88^*^
Play with other children outside of home and school	18 (12)	23 (15)	50 (33)	45 (30)	14 (9)	42 (32)	7 (5)	1 (1)	0 (0)	134.44^*^
Sporting event of child	14 (10)	24 (16)	35 (24)	40 (27)	34 (23)	40 (31)	18 (14)	3 (2)	0 (0)	112.07^*^
Eating at a restaurant	13 (9)	19 (13)	45 (30)	52 (34)	22 (15)	65 (49)	17 (13)	3 (2)	0 (0)	122.03^*^
Movies/theatre	13 (9)	21 (14)	39 (26)	29 (19)	47 (32)	55 (42)	10 (8)	6 (5)	4 (3)	110.78^*^
Religious service, event, or education	16 (11)	19 (13)	28 (19)	43 (29)	43 (29)	55 (42)	9 (7)	3 (2)	1 (1)	128.57^*^
Library activity	15 (10)	29 (19)	33 (22)	45 (30)	29 (19)	47 (36)	9 (7)	3 (2)	0 (0)	121.23^*^
Vacations	16 (11)	22 (15)	48 (32)	50 (33)	14 (9)	41 (31)	32 (24)	3 (2)	0 (0)	86.03^*^
Sporting event of another	11 (7)	26 (18)	40 (27)	37 (25)	34 (23)	52 (40)	11 (8)	1 (1)	2 (2)	125.51^*^
Community event	14 (9)	25 (17)	43 (28)	45 (30)	24 (16)	63 (48)	11 (8)	5 (4)	1 (1)	108.72^*^
Play at the playground/park	33 (22)	32 (21)	52 (34)	31 (21)	3 (2)	40 (30)	5 (4)	0 (0)	0 (0)	97.11^*^
Public restroom	19 (13)	29 (19)	31 (21)	53 (36)	17 (11)	62 (47)	18 (14)	8 (6)	2 (2)	67.64^*^
Public transportation	23 (15)	29 (19)	37 (25)	39 (26)	22 (15)	37 (28)	12 (9)	9 (7)	7 (5)	49.15^*^
**Healthcare scale**										
Dental appointments	4 (3)	21 (14)	33 (22)	67 (44)	26 (17)	51 (39)	24 (18)	14 (11)	1 (1)	101.26^*^
Doctor appointments	6 (4)	24 (16)	40 (26)	68 (45)	13 (9)	58 (44)	24 (18)	12 (9)	0 (0)	92.71^*^

*Response categories reflect level of parental effort: 1 = none, 2 = a little, 3 = some, 4 = a lot, 5 = too much to participate; X^2^ = chi square test of independence, df = 4;*

* *p < 0.0001*.

Fifteen PES items assessed the level of parental effort required to enable children to participate in four types of home-based activities: dressing (three items: e.g., putting on socks and shoes), hygiene/self-care (six items: e.g., bathing, toileting), sleeping (two items: falling and staying asleep), and social/play activities (4 items: mealtime with family, play with other children). The 19 remaining PES items assessed the level of effort required for parents to help their children participate in three types of community activities: social activities (five items, e.g., parties for another child, meals at home of family or friends), community outings (12 items, e.g., movies/theaters, library activity, using a public restroom), and receipt of healthcare services (two items: dentist and doctor appointments/procedures).

#### Caregiver Strain Questionnaire

A randomly selected subset of 135 parents (49 parents of children with ASD, 86 parents of neurotypical children) completed the Caregiver Strain Questionnaire (CGSQ). CGSQ responders and non-responders were alike on all demographic characteristics. The CGSQ overall strain score reflects objective (e.g., disruption of personal time, financial strain, effect on work), internalized (e.g., worry, guilt, and unhappiness), and externalized (e.g., negative feelings directed toward the child, relating poorly with child) experiences of caregiver burden (14). The CGSQ has strong reliability and validity evidence for families of children with ASD (15).

### Procedures

All measures were administered to parents online using Qualtrics Survey Software. A randomly selected subset of participants (*n* = 126) completed the PES again approximately two weeks after initial administration to enable evaluation of the measure's test-retest reliability.

### Data Analysis

Response category frequencies and test-retest reliability (intraclass correlation coefficient; ICC) were calculated for each item. Chi square tests of independence were conducted to compare item response category distributions between the ASD and neurotypical subgroups. We expected to observe shared variance within subsets of PES item that reflected similar or related activities (e.g., dressing). We also expected parental effort to be correlated across activity subtypes, reflecting a higher-order effort dimension. To test these assumptions, we fit a 2-level confirmatory factor analysis (CFA) model to the data using the lavaan package in R (16). The model specified that each PES item contributed to one of six factors (dressing, hygiene/self-care, sleeping, home-based social/play activities, community-based activities, or healthcare), which themselves contributed to a higher-order *total effort* factor. We evaluated model fit for the full sample using three indices: Comparative Fit Index (CFI ≥ 0.95), Tucker–Lewis fit index (TLI ≥ 0.95), and Root Mean Square Error of Approximation (RMSEA ≤ 0.08) (17). Assuming adequate model fit, items with loadings ≥ 0.70 were thought to contribute to the measurement of parent effort (18, 19).

Next, we fit separate unidimensional graded response models to PES item response data for each scale to estimate item response theory (IRT) discrimination and difficulty parameters (20). The discrimination statistic (*a*) measures the degree to which an item differentiates respondents by their level of the latent trait (e.g., parental effort for dressing). The IRT model also produces threshold parameters (*b*). For a measure with five response categories, the IRT model produces four item threshold statistics (b_1_-b_4_). Each *b* parameter indicates the level of effort required of parents who endorse a specific response to a particular item (e.g., “some effort is required to assist children in putting on socks and shoes”).

Items were selected for inclusion in the final PES scales based on factor analytic and IRT analyses. Internal consistency and test-retest reliability of the final PES scales were evaluated using Cronbach's α and ICCs, respectively. Once scale composition was finalized, we calculated PES scale scores for each respondent using Bayesian Expected A Posteriori (EAP) estimation (21), which uses an individual's pattern of responses and IRT parameters to estimate standardized theta scores. The theta scores for subscales were linearly transformed to standardized scores with a mean of 10 and a standard deviation of 3 as follows: (Θ*3)+10. We calculated a total PES scoring by summing scale scores and transforming the sum score to standardized T-scores (*M* = 50, *SD* = 10). We compared PES scores between the ASD and neurotypical subgroups using independent 2-group *t*-tests.

To evaluate construct validity of the PES, we tested for expected score differences between ASD and neuro-typical groups using generalized lineal models. For children with ASD, we used Pearson correlation to evaluate associations between PES scores and ASD symptoms as measured by the GARS Autism Index. We hypothesized that parents of children with ASD would report higher levels of effort and that ASD symptom severity would be positively associated with parental effort. Finally, we explored associations between parent effort and caregiver strain by estimating correlations between PES scores and the CSQ Global Strain score. We expected to observe moderate associations, because parent effort and caregiver strain are related, but distinct constructs.

## Results

Overall, the sample included a higher proportion of wealthier families than exist in the general US population. The majority of families (73%) had annual incomes >$60,000 and 59% earned more than $80,000 annually. Families of children with ASD and those with neurotypical children did not differ on any socio-demographic characteristic, except child gender (ASD: 76% boys, NT: 53% boys). Additional sociodemographic information is shown in Table 1.

PES item descriptive statistics are shown in Table 2. Compared to parents of neurotypical children, parents of children with ASD reported significantly higher effort levels on all PES items. For the ASD subgroup, the most effortful activities included dental appointments, attending religious services, and going to the movies or theater. Parents of neurotypical children rated hair washing and bathing as the most effortful activities. For both subgroups, playing with toys or objects was the least effortful activity for parents.

In an initial version of the two-level CFA model, 4 of the 34 items had low factor loadings (<0.70). These items assessed the parental effort required for hair care, nail care, swimming, and going to an amusement park. These items were removed. We addressed local dependency for a single item pair (child party, parent party) by removing the later item. The two-level CFA model was an excellent fit for the remaining 29 items (CFI = 0.97, TLI = 0.96, RMSEA = 0.08). Items that contributed to each subscale were internally consistent. Cronbach's alpha statistics for the subscales ranged from 0.81 to 0.95 (Table 3). Test-retest reliability was moderate or good for all PES items (ICCs: 0.51–0.82) and scales (ICCs: 0.61–0.86) (22).

**Table 3:** Parent Effort Scale (PES) scale statistics.

	**Factor** **loading^a^**	**Descriptive and reliability statistics**									**ASD vs.** **neurotypical** **comparison^b^**
		**Total sample** **(*N* = 304)**			**ASD sample** **(*n* = 165)**			**Neurotypical sample** **(*n* = 139)**			
**PES scales**		**M (SD)**	**α**	***ICC***	**M (SD)**	**α**	***ICC***	**M (SD)**	**α**	***ICC***	***t***
**Subscales**											
Dressing	0.88	10.13 (2.99)	0.91	0.73	11.77 (2.58)	0.86	0.55	8.33 (2.29)	0.85	0.72	11.88^*^
Hygiene/self-care	0.82	9.97 (2.77)	0.82	0.70	11.12 (2.72)	0.78	0.64	8.69 (2.11)	0.72	0.52	8.46^*^
Sleep	0.62	9.96 (2.66)	0.81	0.61	10.81 (2.77)	0.79	0.57	8.96 (2.21)	0.77	0.60	6.24^*^
Home social	0.80	9.95 (2.78)	0.87	0.68	11.34 (2.52)	0.80	0.46	8.31 (2.04)	0.80	0.49	11.14^*^
Community participation	0.87	9.71 (3.41)	0.96	0.84	11.98 (2.60)	0.92	0.68	7.14 (2.14)	0.90	0.59	17.20^*^
Healthcare	0.89	10.02 (2.85)	0.89	0.83	11.63 (2.30)	0.78	0.76	8.31 (2.30)	0.89	0.71	12.39^*^
Total scale	–	50.00 (10.00)	0.87	0.86	56.49 (7.80)	0.78	0.70	42.64 (6.18)	0.72	0.75	16.65^*^

a *Scale-to-total loadings.*

b *Comparison of PES subscale scores between ASD and neurotypical groups using independent 2-group t-test; α, Cronbach's alpha (internal consistency); ICC, intraclass correlation coefficients (test-retest reliability); ICC samples: total sample (N = 128), ASD subsample (n = 63), NT subsample (n = 65);*

* *p < 0.0001*.

**Figure 1:**
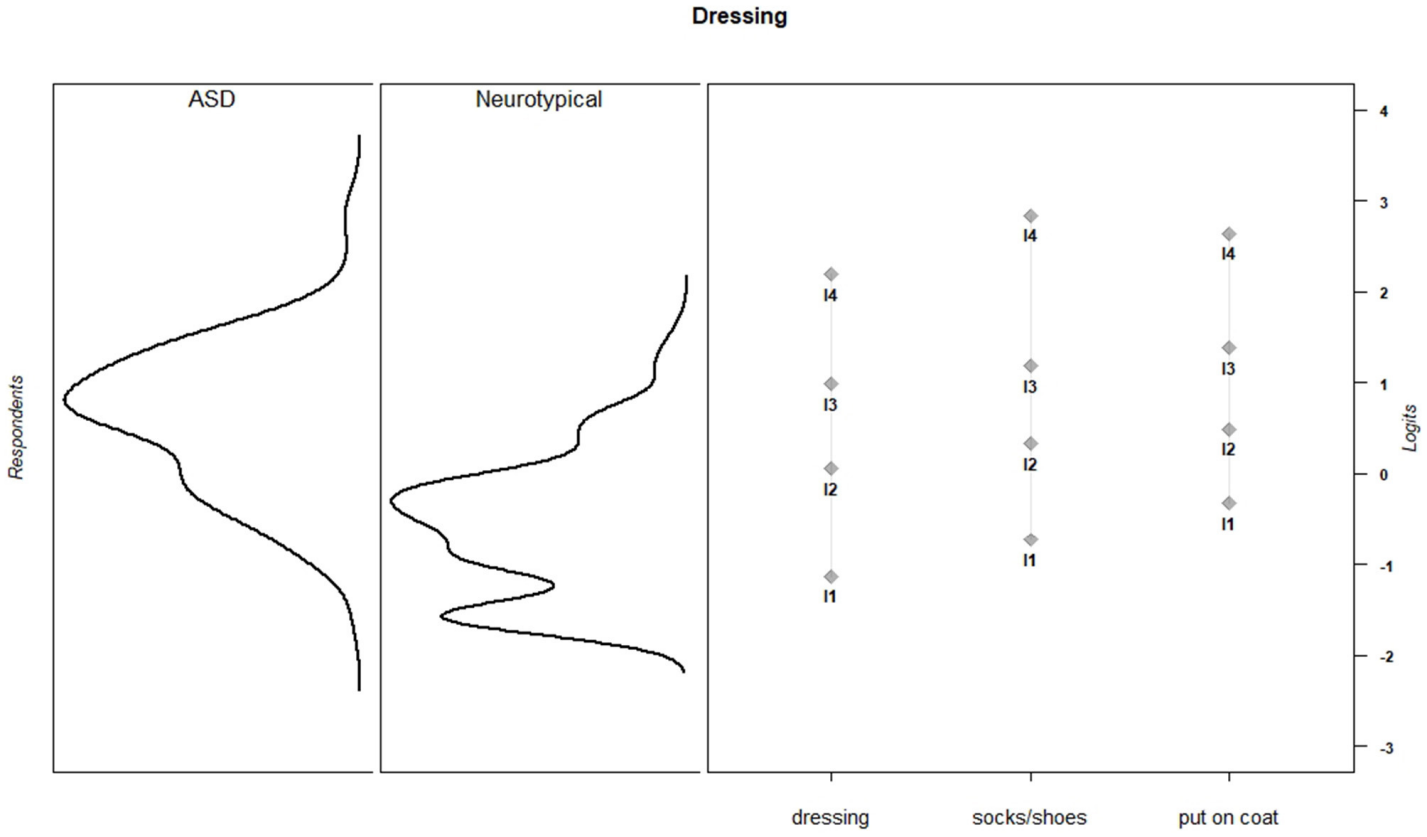
Dressing item parameters and person parameters by subgroup.

**Figure 2:**
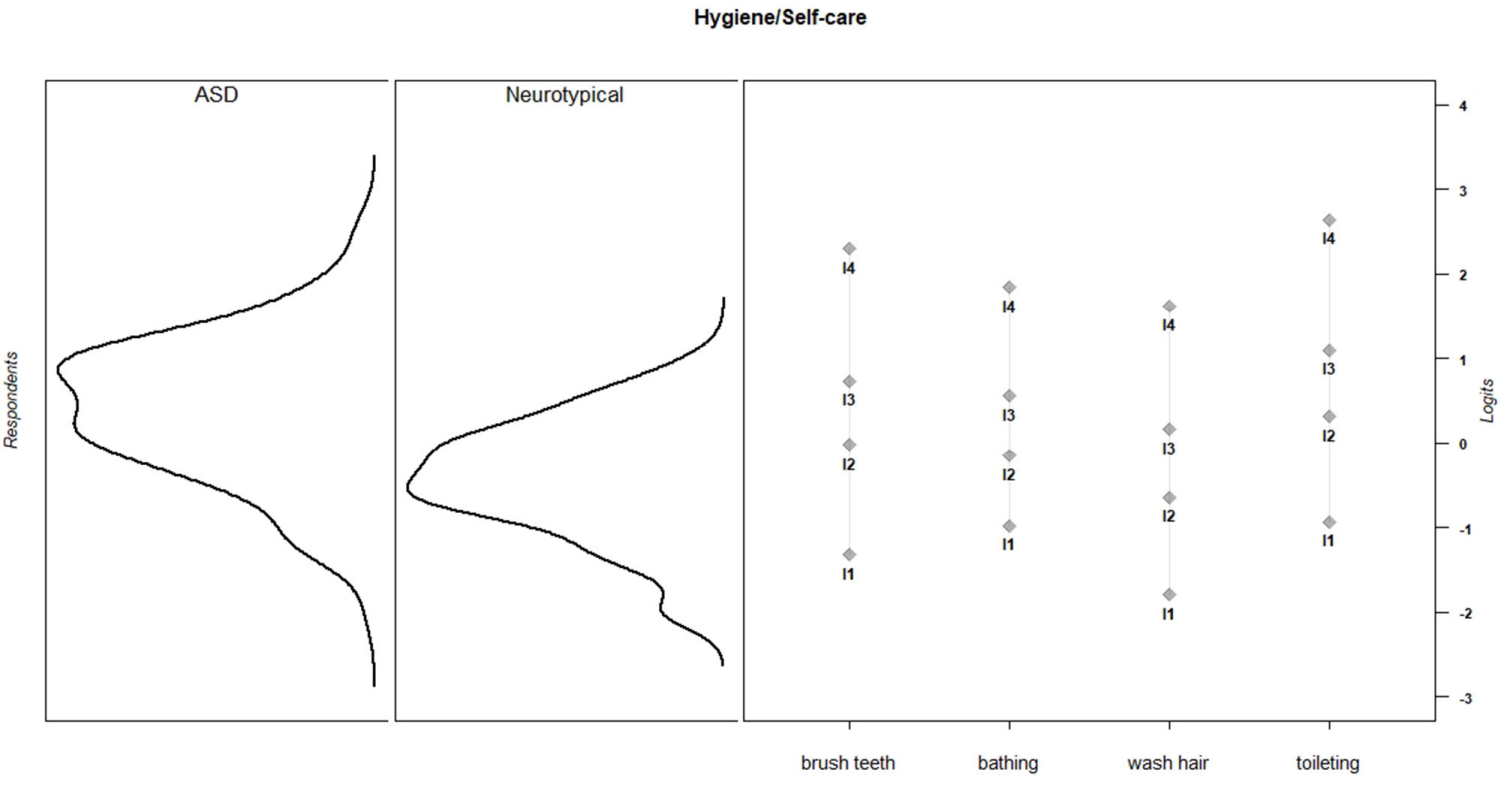
Hygiene/self-care item parameters and person parameters by subgroup.

**Figure 3:**
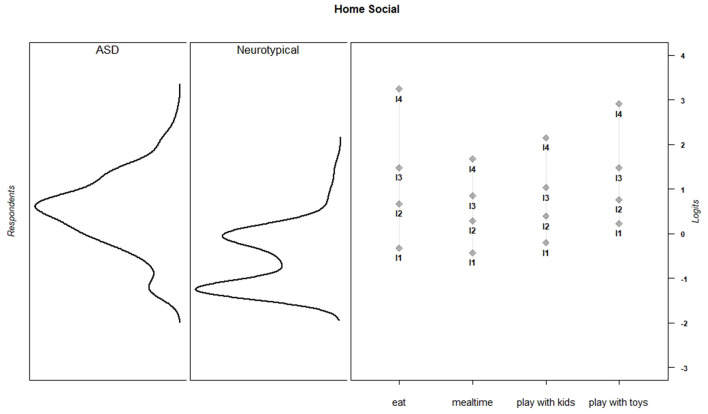
Home social item parameters and person parameters by subgroup.

**Figure 4:**
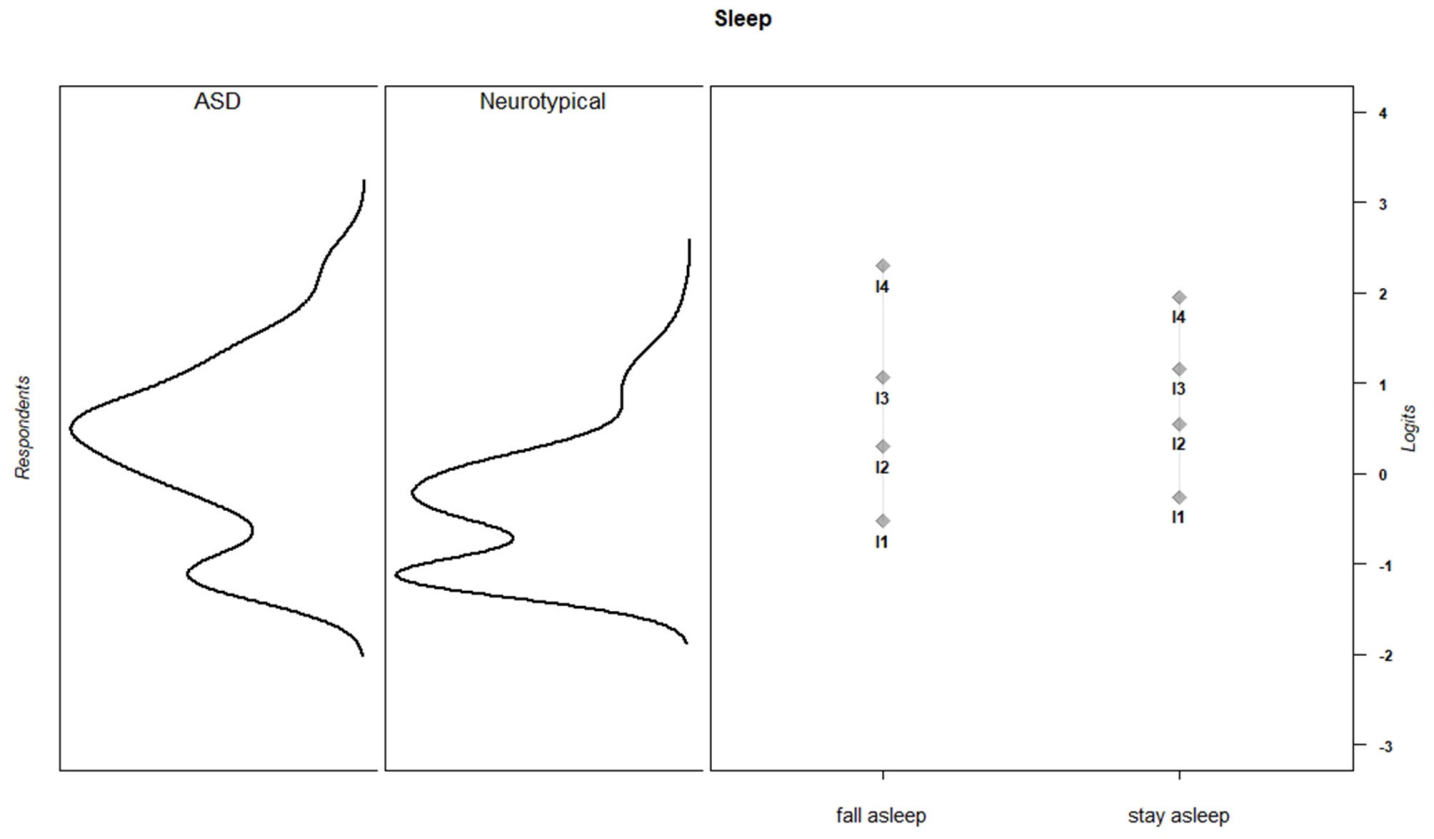
Sleep item parameters and person parameters by subgroup.

**Figure 5:**
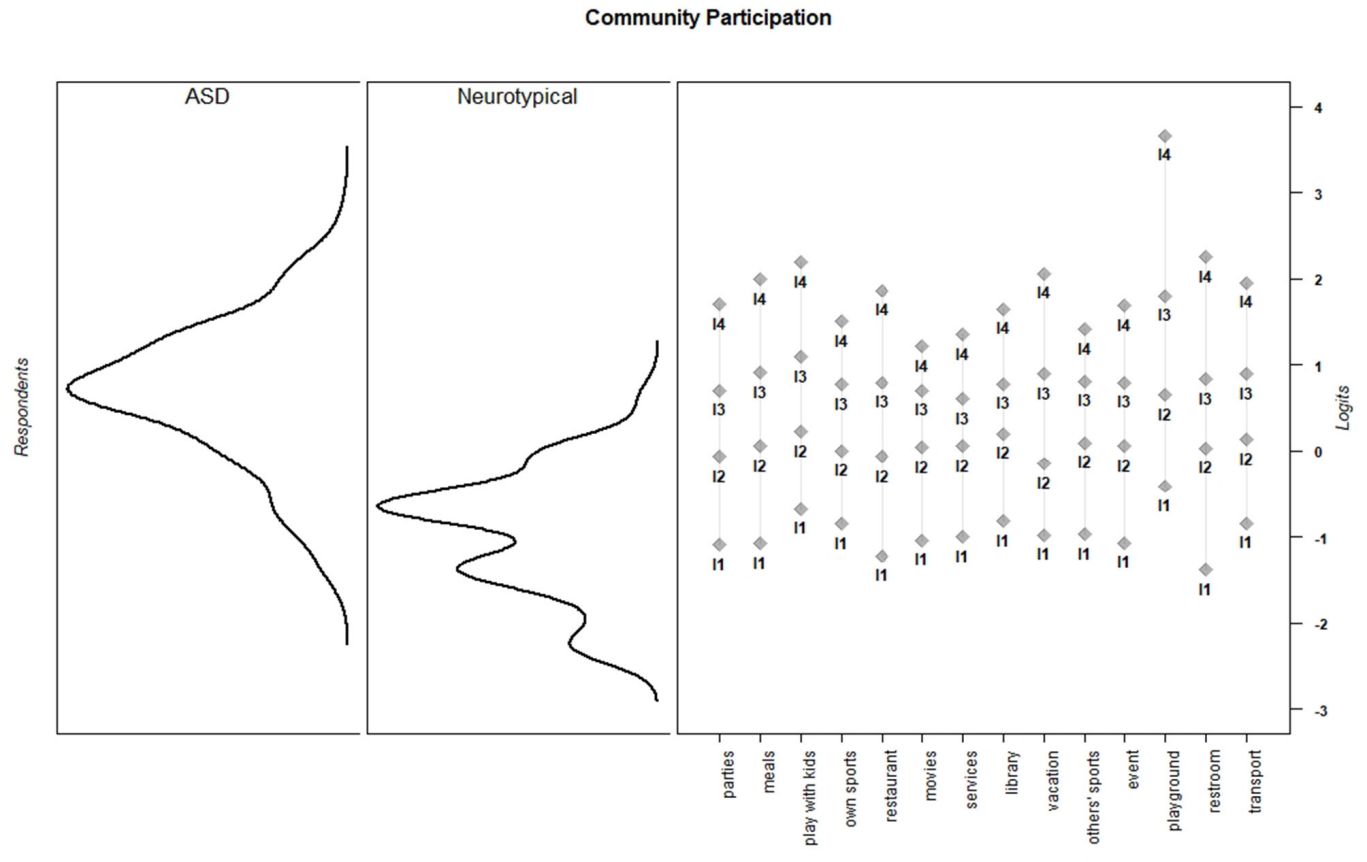
Community participation item parameters and person parameters by subgroup.

**Figure 6:**
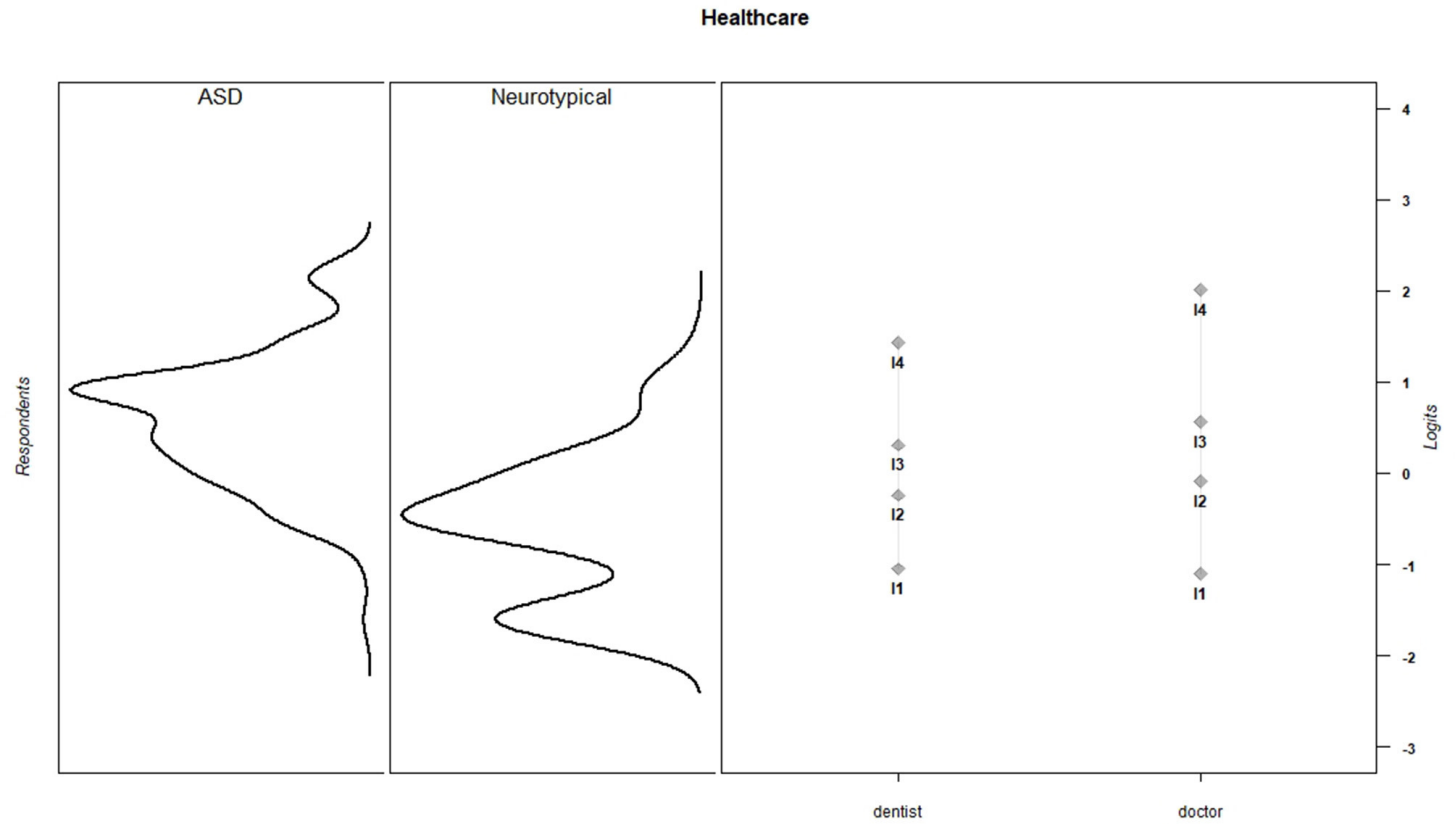
Healthcare item parameters and person parameters by subgroup.

Discrimination and threshold parameters from the graded response models are shown in Table 4. In the current sample, each PES item discriminated among the varying levels of effort needed to support children's activity participation (*M* = 2.66, *SD* = 0.73, range = 1.50–3.96). For all items, category thresholds were ordered as expected. The probability of endorsing higher response categories increased as the level of parental effort increased. Item-person maps for each PES scale show that in the current sample, items assessed a wide range of parental effort levels, but the items provided more information about children for whom participation requires moderate to high levels of parental effort (Figures 1–6).

**Table 4:** Parent Effort Scale (PES) item statistics.

**Scale/Item**	**Factor loading**	**Test-retest reliability**	**IRT parameters**				
		**(*ICC*)**	***a***	***b_1_***	***b_2_***	***b_3_***	***b_4_***
*N*	304	128			304
**Dressing scale**							
Dressing (excluding socks/shoes/coat)	0.91	0.71	3.66	−1.13	0.05	0.98	2.19
Putting on socks/shoes	0.89	0.66	2.89	−0.73	0.33	1.18	2.84
Putting on coat by self or another	0.95	0.66	2.81	−0.33	0.49	1.38	2.64
**Hygiene/self-care scale**							
Tooth brushing	0.84	0.67	1.97	−1.32	−0.02	0.73	2.30
Bathing (excluding washing hair)	0.80	0.54	3.79	−0.98	−0.15	0.56	1.84
Washing hair	0.65	0.56	2.30	−1.79	−0.64	0.16	1.62
Toileting (diaper changes/using toilet)	0.86	0.71	1.50	−0.93	0.31	1.10	2.64
**Sleep scale**							
Falling asleep	0.84	0.61	2.83	−0.53	0.30	1.06	2.30
Staying asleep through night	0.91	0.64	3.75	−0.26	0.55	1.16	1.95
Home social scale							
Eat	0.79	0.59	3.30	−0.32	0.67	1.48	3.24
Mealtime with family members	0.91	0.66	3.86	−0.43	0.29	0.85	1.67
Play with other children in home	0.93	0.58	2.30	−0.21	0.39	1.03	2.15
Play with toys/objects	0.86	0.51	1.92	0.23	0.76	1.47	2.91
**Community participation**							
parties for another child in community/at another's home	0.89	0.80	2.77	−1.09	−0.07	0.70	1.70
meals at family or friends' home	0.88	0.77	2.60	−1.08	0.05	0.91	1.99
Play with other children outside of home and school	0.84	0.72	2.12	−0.67	0.22	1.09	2.19
Sporting event of child	0.86	0.61	2.37	−0.85	0.00	0.77	1.50
Eating at a restaurant	0.85	0.72	2.55	−1.22	−0.07	0.79	1.86
Movies/theater	0.84	0.74	2.28	−1.04	0.04	0.70	1.21
Religious service, event, or education	0.84	0.76	2.23	−1.00	0.05	0.60	1.35
Library activity	0.86	0.69	2.55	−0.82	0.20	0.78	1.65
Vacations	0.85	0.64	2.46	−0.98	−0.15	0.89	2.06
Sporting event of another	0.90	0.71	3.15	−0.97	0.09	0.81	1.41
Community event	0.89	0.66	2.91	−1.07	0.05	0.79	1.69
Play at the playground/park	0.79	0.69	1.59	−0.41	0.65	1.80	3.66
Public restroom	0.76	0.62	1.59	−1.38	0.03	0.83	2.25
Public transportation	0.72	0.58	1.54	−0.84	0.13	0.89	1.95
**Healthcare scale**							
Dental appointments	0.91	0.82	3.96	−1.05	−0.24	0.31	1.43
Doctor appointments	0.95	0.76	3.49	−1.10	−0.09	0.56	2.01

*ICC, intraclass correlation coefficient; IRT, item response theory; IRT parameters: a = discrimination; b_1–4_ = difficulty*.

**Figure 7:**
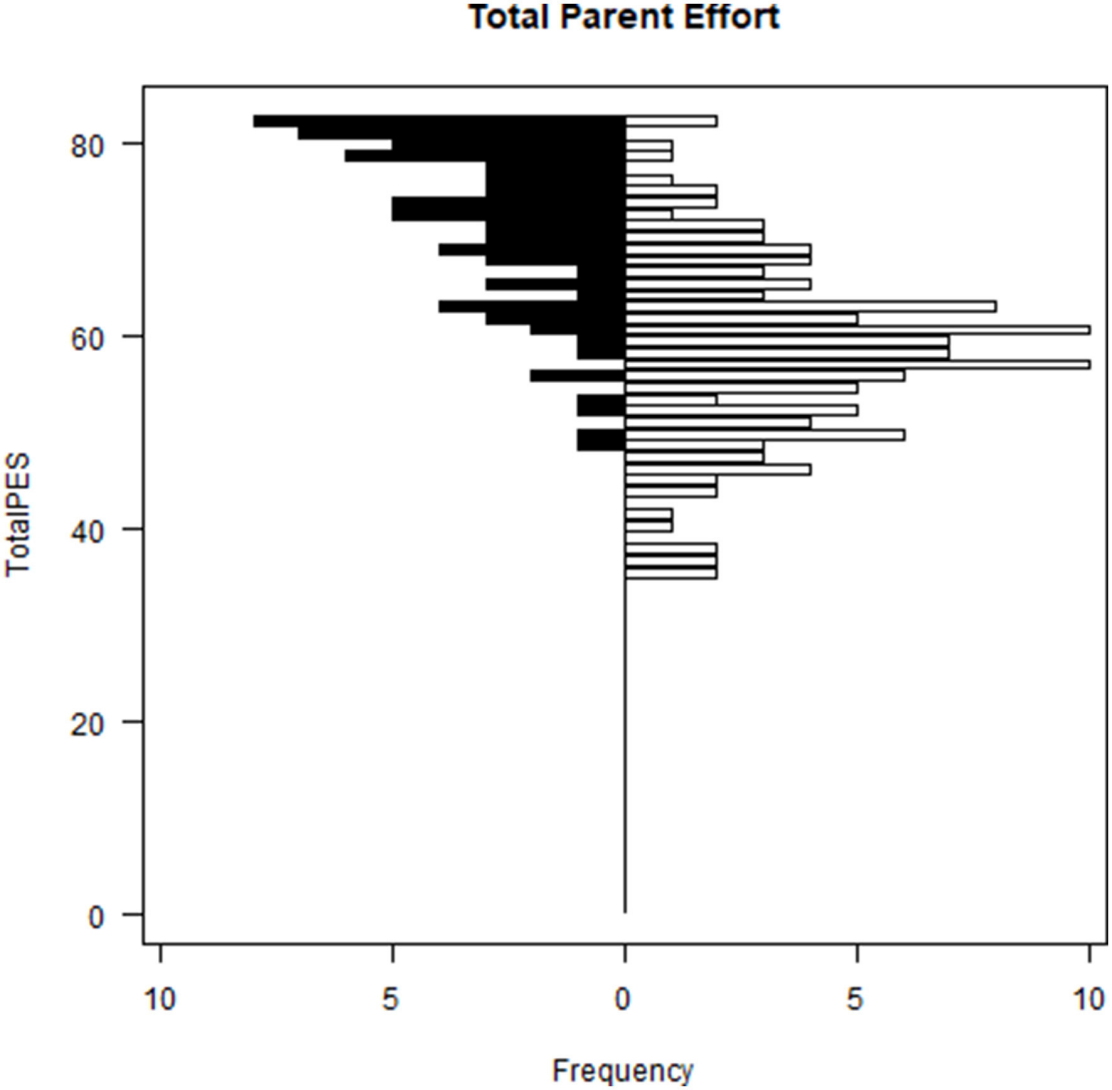
Parent effort scale distributions—total parent effort: children with ASD vs. NT children. *F*_(1, 281)_ = 268.8, *p* < 0.0001; Black bars = children with ASD, white bars = NT children.

Parents of children with ASD had significantly higher PES subscale and total scale scores than parents of neurotypical children (Table 3; Figure 7). ASD symptom severity, as measured by the GARS Autism Index, was positively associated with all PES subscale scores (range: 0.27−0.49) and PES Total (0.60) (Table 5). PES subscale scores are moderately correlated with the CSQ Total: Global Strain score (range: 0.49–0.69; Table 5).

**Table 5:** PES concurrent validity.

	**PES scale scores**						
	**Dressing**	**Hygiene/Self-care**	**Sleep**	**Home social**	**Community participation**	**Healthcare**	**Total**
GARS scores^a^ Total: Autism Index	0.49	0.47	0.27^*^	0.38	0.45	0.41	0.60
Caregiver Strain Questionnaire^b^ Total: Global strain	0.53	0.40	0.60	0.69	0.67	0.49	0.72

a *N = 167 child with ASD.*

b *N = 133 (ASD: n = 48; NT: n = 85); all correlations statistically significant, p < 0.001, expect*

* *p < 0.01*.

## Discussion

The purpose of this study was to refine and evaluate the psychometric properties of the PES, a caregiver-report measure of the parent effort needed to enable children's participation in home- and community-based activities. Similar to the International Classification of Functioning, Disability and Health (ICF) construct of participation, the PES defines participation as involvement in a life situation (23). At this level, the child is involved in the activity; however, the PES measures the environmental factor of parental effort required to support participation in that activity rather than the capacity of the child to complete the task (23). The PES measures parental effort in six domains: (1) dressing, (2) hygiene/self-care, (3) sleep, (4) social activities in the home, (5) social activities in the community, and (6) healthcare. Subscale scores are combined to generate a total score. CFA modeling provided support for the tool's structural validity after four items were removed. These included two personal hygiene items: “hair care” and “nail care.” Two additional items, “swimming” and “going to an amusement park” were removed because they failed to contribute to the measurement of social events in the community, perhaps because they are seasonal in nature. Another item, “family parties at another's home” was removed because it provided nearly identical information as “parties for another child in the community/at another's home” in this sample. The final PES scales were found to have adequate internal consistency and test-retest reliability. The final PES subscales and total score are reliable and precise.

As expected, parents of children with ASD reported expending significantly more effort than parents of neurotypical children and ASD symptom severity was positively associated with effort levels. For children with ASD, language delays (24), sensory sensitivities (25), sleep differences (26), and behavioral rigidity and maladaptive behaviors (27) may increase parent effort demands. When parents of children with ASD perceive that they are unable to manage these challenging behaviors, they may experience internalized stigma and further restrict their child's participation opportunities for fear of others' judgement (28). For parents of children with ASD, effort demands are greater for community-based activities than for activities done at home (e.g., dressing, hygiene, sleep, and home-based social activities). Due to this, parents must often weigh the cost-benefit of their child's participation in home and community activities (29). Family with children with ASD may participate in fewer activities outside the home because the gap in child-environment fit is greater. Community contexts are less predictable and environmental modifications to support children's participation are more challenging to implement (10).

Parent effort and caregiver strain were found to be moderately correlated, indicating that they are related, but distinct constructs. Whereas parental effort describes the amount of perceived work required to complete caregiving tasks, strain describes a perceived burden of more responsibilities and demands that result in negative psychological consequences (14). Parents who expend high levels of effort across multiple activity domains for prolonged periods of time will likely experience strain (30). In contrast, parents who expend moderate (and gradually less) effort to facilitate their child's participation in a one or a small number of domains may view the effort as rewarding and a source of pride (31).

By gauging the level of parent effort within and across activity domains and over time, the PES can be used to target and evaluate the effectiveness of interventions that aim to help parents enhance their child's participation and thereby, support children's cognitive and social development. PES subscales can be used to create a “*parent effort profile*” that distinguishes activity domains that require minimal parent effort (strengths) from those that demand significant effort (challenges). Providers could work with families to identify child or parent skills (or skill deficits) and environmental conditions that reduce parent effort requirements for some domains and increase effort demand for others. In this way, PES results can inform the design of interventions that ease parental effort and thus, increase opportunities for children to participate. When administered repeatedly, the PES could be used to gauge the effectiveness of these interventions.

Limitations of this study include participant homogeneity and sampling bias. The sample over represents wealthier, Caucasian, and suburban families whose participation opportunities and preferences may differ from those of less well-resourced, urban, or rural families and those with different cultural backgrounds. For example, families in this study may have more opportunities to participate in costly community leisure activities and less need for public transportation. The PES should be further validated in larger and more diverse samples, including parents of children with other neurodevelopmental and chronic health conditions that influence parenting effort and children's participation.

## Data Availability Statement

The raw data supporting the conclusions of this article will be made available by the authors, without undue reservation.

## Ethics Statement

The studies involving human participants were reviewed and approved by Temple University IRB. The patients/participants provided their written informed consent to participate in this study.

## Author Contributions

All authors contributed to the conception and design, acquisition of data, or analysis and interpretation of data. They all drafted and revised the manuscript's important intellectual content.

## Conflict of Interest

KB is employed at Janssen Pharmaceuticals Inc., and AP owns and is employed by Piller Child Development, LLC. The remaining authors declare that the research was conducted in the absence of any commercial or financial relationships that could be construed as a potential conflict of interest.

## Publisher's Note

All claims expressed in this article are solely those of the authors and do not necessarily represent those of their affiliated organizations, or those of the publisher, the editors and the reviewers. Any product that may be evaluated in this article, or claim that may be made by its manufacturer, is not guaranteed or endorsed by the publisher.
